# Eda controls the size of the enamel knot during incisor development

**DOI:** 10.3389/fphys.2022.1033130

**Published:** 2023-01-09

**Authors:** Lucie Horakova, Linda Dalecka, Oldrich Zahradnicek, Katerina Lochovska, Herve Lesot, Renata Peterkova, Abigail S. Tucker, Maria Hovorakova

**Affiliations:** ^1^ Institute of Histology and Embryology, 1st Faculty of Medicine, Charles University, Prague, Czechia; ^2^ Department of Cell Biology, Faculty of Science, Charles University, Prague, Czechia; ^3^ Department of Radiation Dosimetry, Nuclear Physics Institute, Czech Academy of Sciences, Prague, Czechia; ^4^ First Department of Medicine—Department of Hematology First Faculty of Medicine, Charles University and General University Hospital in Prague, Prague, Czechia; ^5^ Laboratory of Odontogenesis and Osteogenesis, Institute of Animal Physiology and Genetics, Academy of Sciences, Brno, Czechia; ^6^ Department of Histology and Embryology, 3rd Faculty of Medicine, Charles University, Prague, Czechia; ^7^ Department of Craniofacial and Regenerative Biology, King´s College London, Guys Hospital, London, United Kingdom

**Keywords:** tabby mouse, mouse incisor, shh expression, rudiment, tooth development

## Abstract

*Ectodysplasin* (*Eda*) plays important roles in both shaping the developing tooth and establishing the number of teeth within the tooth row. *Sonic hedgehog* (*Shh*) has been shown to act downstream of *Eda* and is involved in the initiation of tooth development. *Eda−/−* mice possess hypoplastic and hypomineralized incisors and show changes in tooth number in the molar region. In the present study we used 3D reconstruction combined with expression analysis, cell lineage tracing experiments, and western blot analysis in order to investigate the formation of the incisor germs in *Eda−/−* mice. We show that a lack of functional Eda protein during early stages of incisor tooth germ development had minimal impact on development of the early expression of Shh in the incisor, a region proposed to mark formation of a rudimental incisor placode and act as an initiating signalling centre. In contrast, deficiency of Eda protein had a later impact on expression of *Shh* in the primary enamel knot of the functional tooth. *Eda−/−* mice had a smaller region where *Shh* was expressed, and a reduced contribution from Shh descendant cells. The reduction in the enamel knot led to the formation of an abnormal enamel organ creating a hypoplastic functional incisor. *Eda* therefore appears to influence the spatial formation of the successional signalling centres during odontogenesis.

## Introduction

EDA is a member of the tumour necrosis factor (TNF) superfamily and plays a role in the development of all ectodermal organs, including teeth, sweat glands and hair. The *Eda* pathway consists of EDA, its receptor EDAR, and a cytosolic adaptor molecule EDARADD (Edar-associated death domain) (Mikkola, 2008). *Eda* plays a key role in the formation of ectodermal placodes specifying the initiation of organogenesis (Mustonen et al., 2004), and later playing a role in shaping organs (Cui and Schlessinger, 2006; Fons Romero et al., 2017). The *Eda* signalling pathway is regulated by several signalling pathways (SHH, FGF, WNT, BMP), and reciprocally regulates the expression of a number of pathways, providing spatio-temporal cues during ectodermal appendage formation.

*Sonic hedgehog* (*Shh*) has been described as acting downstream of *Eda* (Cui et al., 2014). *Shh* is involved in odontogenesis (Bitgood and McMahon, 1995; Cobourne and Sharpe, 2010) and participates in both lateral (epithelial-mesenchymal) and planar (epithelial-epithelial) signalling during early tooth development (Hardcastle et al., 1998). *Shh* promotes tooth placode invagination on the way to bud formation (Li et al., 2016), regulates growth, and determines the shape of the tooth (Dassule et al., 2000).

Using 3D reconstructions combined with *Shh* whole mount *in situ* hybridization (WISH), it has been shown that there are two serially appearing areas of *Shh* expression present during development of the lower incisors in wildtype (WT) mice. The first is superficially placed while the later forms deeper within the epithelium, corresponding to the primary enamel knot of the functional mouse incisor (Hovorakova et al., 2011, 2013, 2016; Ahtiainen et al., 2016). The enamel knot is a morphological structure (Butler, 1956; Lesot et al., 1996) that houses a signalling centre, with different signalling pathways having nested patterns of expression (Jernvall et al., 1994). The early superficial region of expression has been suggested to mark a rudimentary (so called milk or prelacteal) incisor placode, and has been associated with having a determinative function controlling patterning in the incisor area (Hovorakova et al., 2011). The activity of the ectodysplasin/Edar/nuclear factor κB pathway is found in the early superficial signalling centre, and its experimental inactivation leads to the formation of a smaller incisor bud (Ahtiainen et al., 2016).

Hypohidrotic ectodermal dysplasia (HED, OMIM 224900) is a rare autosomal recessive syndrome resulting from germline mutations in any of the *EDA, EDAR* or *EDARADD* genes in humans (Kere et al., 1996; Arte et al., 2013). HED is characterized by defective development of multiple ectodermal appendages leading to sparse hair, absent or severely reduced sweating and missing or misshapen deciduous and permanent teeth (for review see: Lefebvre and Mikkola, 2014). The *EDA* gene is on the X chromosome, while *EDAR* and *EDARADD* are autosomal.

The Tabby (Ta) mouse has a naturally occurring mutation in *Eda* and is, therefore, homologous to human X-linked hypohidrotic ectodermal dysplasia (OMIM305100) (Weeks and Blecher, 1983; Blecher, 1986; Srivastava et al., 1997). In adult Tabby mice (*Eda−/−*), the incisors may be hypoplastic and hypomineralized, or fused or absent (Gruneberg, 1965). The defects in enamel exhibit considerable variability (Risnes et al., 2005; Sehic et al., 2009). Morphogenetic studies have shown that deficiency in the *Eda* gene leads to lower incisors with a smaller size, and changes in the shape, position and cyto-differentiation in 100% of cases (Miard et al., 1999). However, the interplay between *Eda* and *Shh* signalling during early development of the mouse incisors is still not fully understood.

The present study focused on the early events during lower incisor development in mice with *Eda* deficiency. Using 3D computer aided reconstructions combined with the visualisation of *Shh* expression by WISH, we performed a detailed longitudinal study of the development of dental and adjacent oral epithelia in the embryonic anterior mandible from ED12.5 to ED15.5 in *Eda*−/− and *Eda*+/+ mice. Immunohistochemistry and western blot analysis was used to determine the reciprocal activity of Shh and Eda proteins during incisor development. Furtherly, a transgenic tamoxifen inducible Cre-loxP system was utilised to trace the cells expressing *Shh* in *Eda*−/− and control mice in order to compare the contribution of Shh expressing cells during later development.

Using this approach, we show that the early initiating superficial *Shh* expressing region appeared normal in *Eda* deficient incisors. However, *Eda* deficiency led to a reduction in the size of the enamel knot and limited the area contributed to by the *Shh* signalling centre resulting in abnormal hypoplastic formation of incisors.

## Material and methods

### Mouse embryos

Eda−/− mice were generated from the inbred Ta strain B6CBACa A^w−J^/A-Eda^Ta^/O (Jackson Laboratory, Maine, United States). The genotype was identified according to external morphological criteria: sparse hair, bald patches behind ears and deformities in the distal portion of the tail for hemizygous males (Eda^Ta/Y^) and homozygous females (*Eda*−/−), striping of the coat for heterozygous females (Eda^Ta/+^). During prenatal stages the genotype is not visually detectable until embryonic day (ED) 14.5, where hair follicles start to be apparent in heterozygous specimens. Based on this, only homozygous females and hemizygous males were used for mating to generate Eda deficient embryos during experiments. *Eda*+/+ embryos on the same background were used as controls. Interestingly this inbred background were smaller than outbred CD1 embryos, highlighting the importance of comparing mice on the same background (Supplementary Image S1). Control *Eda*+/+ mice were mated as a separate line.

In the present study, the *Eda*−/−, and control *Eda*+/+ mice were used for WISH, 3D analysis and western blot. CD1 mice (Charles River, Germany) were used for immunohistochemistry and western blot.

*Eda*−/−*Shh*EGFP mice were generated by crossing *Eda*−/− mice with a transgenic mouse strain B6.Cg-Shhtm1(EGFP/cre) Cjt/J (Jackson Laboratory, Maine, United States) manifesting green fluorescence in *Shh* expressing cells in the offspring. *Eda−/−ShhEGFP+* and control *Eda+/+ShhEGFP* + mice were used for fluorescent microscopy to determine the Shh expression using green fluorescent protein and to supplement the results obtained using WISH with intermediate stages. The mice were genotyped using appropriate Jackson’s Lab Protocols.

C57BL/6mice carrying tamoxifen-inducible Cre fused with the *Shh* allele (B6.129S6-Shh < tm2(cre/ERT2) Cjt*>*/J (Harfe et al., 2004)) and Cre recombinase-sensitive transgenic mice containing *LacZ* (beta-galactosidase) inserted into the *Gt(ROSA)26Sor* locus were crossed with *Eda*−/− mice to get the lines of *Eda* deficient mice for the cell lineage tracing study (Cre-loxP system). The hemizygous males (Eda^Ta/Y^) carrying tamoxifen inducible Cre was reciprocally mated with homozygous females (*Eda*−/−) of the reporter strain. This allowed marking of the cell population expressing *Shh* from the time of the tamoxifen injection into pregnant female mice. In this way a narrow developmental window was generated showing the distribution of labelled cells in the incisor germs in the offspring.

*Eda*+/+ (*Eda*WT) and *Eda+/+Shh*EGFP positive mice were used as controls for performed experiments (Supplementary Image S1). LacZ/ShhERT2cre positive mice were used as controls for the cell lineage tracing experiments.

The mice of relevant strains were mated overnight and the midnight before the morning detection of the vaginal plug was regarded to ED 0.0. The embryos were harvested at ED13.5, 14.5, 14.7, 15.3, 15.5, and 15.7 for fluorescent microscopy, at ED12.5, 13.5, 14.0, 14.5, 15.0, and 15.5 for WISH and 3D analysis, at E13.5 and 14.5 for western blot (WB) and at ED12.5–15.5 for immunohistochemistry. The pregnant mice were sacrificed by cervical dislocation. Immediately after the removing of embryos from uterus, their wet body weight was determined for a refining the chronological staging (Peterka et al., 2002, see also Supplementary Image S1).

### Whole mount *in situ* hybridization (WISH)

To visualize the early odontogenic areas in the mouse lower incisor region, *Shh* expression was detected using WISH in 93 *Eda−/−* and 57 *Eda+/+* mouse embryos. Lower jaws were dissected at ED12.5–15.5, washed in RNase free PBS (pH 7.4) and fixed in 4% paraformaldehyde (PFA) solution overnight at 4°C. Specimens were hybridized according to a standard protocol. The probe for *Shh* was generated by *in vitro* transcription from cDNA fragment (kind gift from Dr. A. McMahon, Harvard University, Cambridge, Massachusetts). The hybridized samples were documented using a stereomicroscope Leica MZ6 connected with a digital camera Leica DC480 (Leica Microsystems GmbH, Wetzlar, Germany) and histologically processed.

Hybridized mouse embryonic heads were embedded in a series of graded solutions of sucrose (Sigma) diluted in PBS (pH 7.4). Then the specimens were embedded in O.C.T. Tissue Tek (Sakura) diluted 1:1 with 20% sucrose, frozen in isopentane (Sigma) cooled on dry ice to -60°C, and sectioned frontally on a cryostat microtome Mikrom HM 560 (Mikrom Walldorf, Germany) in series of 10 μm sections. The sections were post-fixed in 4% paraformaldehyde and counterstained by Nuclear Fast Red (Fluka), dehydrated, and mounted in Neomount (Merck).

### Computer-aided 3D reconstructions

For 3D reconstruction of the epithelium of the developing lower incisor, eight series (3 *Eda−/−* and 5 *Eda+/+*) of stained frontal cryo-sections of hybridized mouse heads representative for *Shh* expression patterns at ED13.5-15.5 were used. The specimens were selected according to the chronological age (ED) refined by a body weight of embryos to create a longitudinal series of successive stages of tooth development (Peterka et al., 2002). In all these specimens, the epithelium of their right and/or left lower incisor area was reconstructed to show the spatial relationship between the morphology of the developing structures and the *Shh* expression. Contours of the dental and adjacent oral epithelium were drawn from each histological section (magnification of: 320x – 500x) using a LEICA DMLB microscope (Leica Microsystems GmbH, Wetzlar, Germany) or a JENAVAL microscope (Carl Zeiss, Jena, Germany) equipped with a drawing chamber. Superimposition of the drawings was performed by the “best-fit method“ with respect to the middle line and to the horizontal level for correct spatial positioning of the reconstructed structures as previously described in Hovorakova et al. (2011). Digitalization of the serial drawings and the correlation of successive images have previously been described (Lesot et al., 1996). Three-dimensional images were generated using VG Studio Max 2.2 software (VG Studio Max, Heidelberg, Germany).

### Epithelial dissociation and fluorescent microscopy

To supplement the results obtained using WISH with intermediate stages, epithelia of the developing lower incisor germs of EGFP positive embryos were dissociated (112 *Eda−/−* and 109 *Eda+/+* mouse embryos were used in total). Incisor regions of the lower jaws were dissected and placed into Hank´s solution. The Hank´s solution was replaced by 1% trypsin solution (Difco Laboratories) at 4°C to dissociate the epithelium from the mesenchyme. Dissociated epithelia were documented in the stop solution (20% FCS in Hank´s solution, Sigma Aldrich) using the inverted fluorescence microscope Leica AF6000 (Leica Microsystems GmbH, Wetzlar, Germany). Shh expression was determined according to the green fluorescence.

### Immunohistochemistry

The rabbit polyclonal SHH [H-160] (sc-9024, Santa Cruz Biotechnology) antibody and goat polyclonal EDA [C17] (sc-18927, Santa Cruz Biotechnology) antibody were used to detect Shh and Eda protein expression, respectively within rostral region of oral epithelium of the lower jaw of CD1 mouse embryos at ED12.5–15.5 (28 embryos were used for Shh expression detection and 26 embryos for Eda). Antigen retrieval was performed for 30 min in Tris-EDTA, pH9 in a water-bath (98°C). Primary antibodies were incubated over night at 4°C in the humidified chamber. The secondary biotinylated goat anti-rabbit (BA-1000, Vector) and rabbit anti-goat (BA-5000, Vector) antibodies in concentration 1:50 and 1:100, respectively, were applied for 1 h at room temperature. The avidin–biotin complex (ABC kit, Vectastain, Vector Laboratories, Burlingame, United States) was applied for 30 min. The expression of proteins was visualised by diaminobenzidine (DAB). Slides were counterstained by alizarin red S. A negative control was processed in the same way except the application of the primary antibody.

### Western blot

Embryos *Eda−/−* for western blot analysis were harvested at ED14.5, what is the relevant stage, where the initiation expression region stops its activity and only posterior enamel knot of the functional incisor is expressing *Shh* (as a control, *Eda+/+* mice at ED13.5 and 14.5 of corresponding developmental stages–with similar body-weights were used). In total 9 *Eda−/−* and 5 *Eda+/+* embryos were used. Lower jaws (without the tongue) were dissected on ice in PBS/Depc and immediately frozen using dry ice. The samples were lysed using 500 ul of lysate buffer. Lysate was mixed with 2x Laemmli buffer 1:1 and the mixture was incubated 10 min in 95°C. Lysed samples (20 ul) were then electrophoretically separated using SDS-PAGE. 5 ul of Prestained protein standard P7712 was used as a marker. Proteins were transferred to Immuno-Blot nitrocellulose membrane or polyvinyldifluoride (PVDF) membrane through 1 h transfer. Then the membrane was blocked for 1 h with 5% milk (T145.2 ROTH, pH 7,2) in PBST or in 1% BSA in PBST for fluorescence. To visualizes corresponding proteins, antibodies anti-SHH (1:250, sc-9024, Santa Cruz Biotechnology) and anti-Actin (1:5000, SAB4301137, Sigma-Aldrich) were used. The membrane was stained overnight in 4°C with primary antibodies, then washed in PBST and stained with secondary antibody (1:3000, A6667, Sigma-Aldrich) for 1 h in room temperature. Membrane was washed in PBST and proteins were visualised using Milipore Immobilon Western Chemiluminescent HRP Substrate or by Li-cor Odyssey 9120 (LI-COR Biotechnology). Resulting images were scanned and protein quantification was performed using VisionCapt software. The analysis was performed in three independent harvestings. The normality of the data was tested by Shapiro-Wilk test and consequently One Way ANOVA test was used to determine the differences of the relative protein levels in *Eda−/−* compared to control *Eda+/+* and WT (CD1) mice.

### Shh expressing cell-lineage tracing - tamoxifen administration and X-Gal staining

Pregnant female reporter mice in the Cre-loxP system (see above) were injected intra-peritoneally with tamoxifen at E11.5, and 12.5 when *Shh* is expressed only in the early anterior initiation signalling centre and at ED13.5, when the anterior *Shh* expressing signalling centre stops its activity and only posterior signalling centre is expressing *Shh* (Hovorakova et al., 2011). Tamoxifen was administrated in a maximal dose of 0.225 mg/g of body weight (Hayashi and McMahon, 2002). Such a concentration is not hazardous for pregnant mice or embryos and is sufficient for the fast activation of recombination. The embryos (58 control LacZ positive embryos and 8 *Eda−/−* LacZ postitive embryos) were harvested at ED14.5 and 15.5 (48/72/96 h respectively) after tamoxifen administration (at the stages, where incisor germs already express *Shh* in their appearing enamel knots). The heads of embryos were washed in a phosphate buffer at 4°C and pre-fixed for 20 min in 4% paraformaldehyde (PFA). The samples were stained in the staining phosphate buffer (X-gal (Sigma) concentration 3 mM) and beta-galactosidase activity was detected by incubation in the dark during night at 37°C. Samples with positive staining were post-fixed in PFA (4%) overnight. After post-fixation, the samples were washed in PBS. The upper and lower jaws were dissected, and the lower jaws were photographed using a Leica MZ6 stereomicroscope equipped with a Leica EC3 digital camera (Leica Microsystems GmbH, Wetzlar, Germany). After photo-documentation, the samples were post-fixed in Bouin solution for a minimum of 2 weeks, and then histologically processed. The X-gal positive samples were routinely embedded in paraffin and 10 μm thick serial frontal sections were prepared. The sections were counterstained with Fast red (Fluka). The stained sections were dehydrated and covered using Neomount (Merck). Histology was documented using Leica DMLB microscope equipped with Leica MC170HD digital camera (Leica Microsystems GmbH, Wetzlar, Germany).

## Results

### Eda and Shh do not overlap in the secondary incisor signalling centre

**FIGURE 1:**
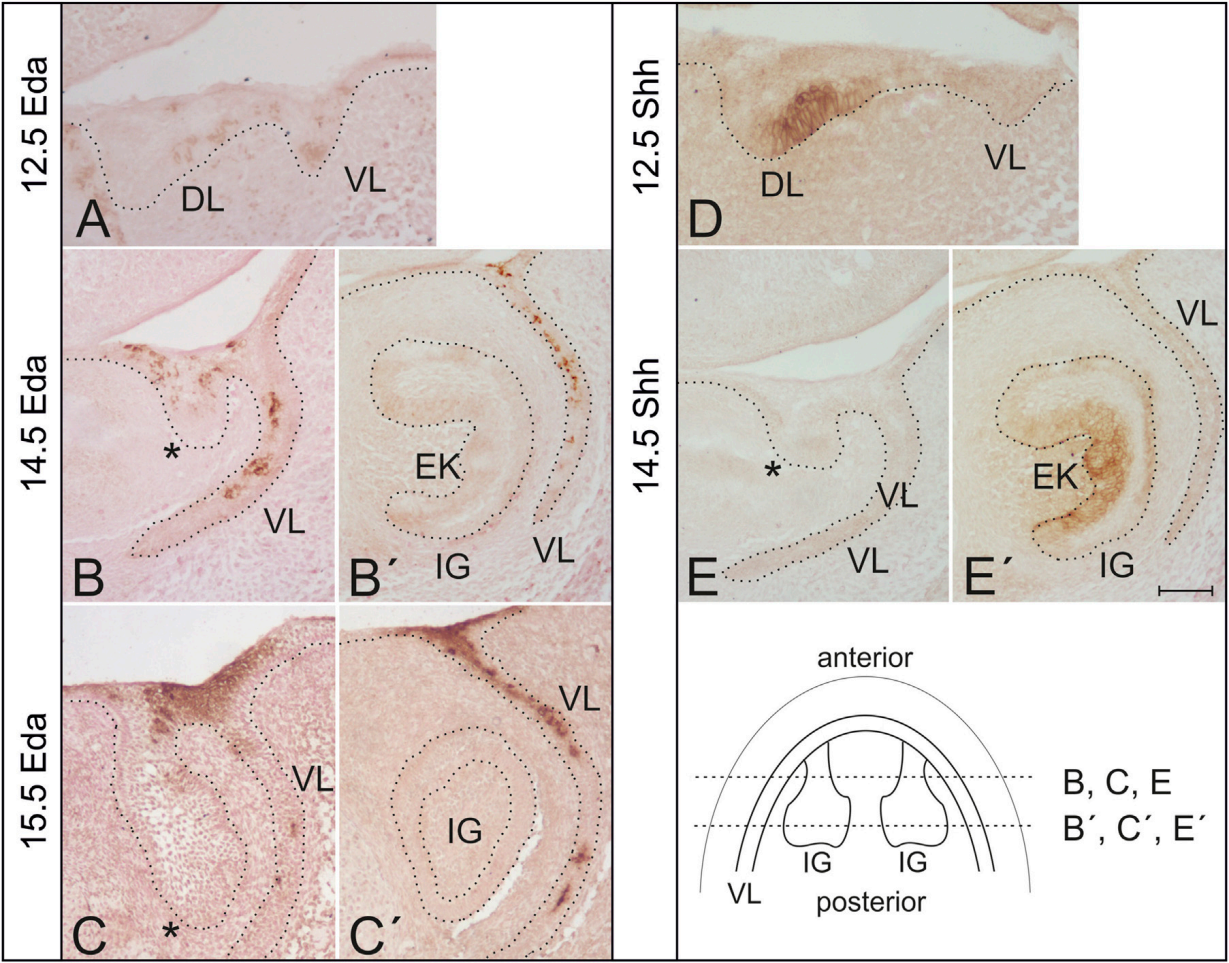
Expression of Eda and Shh protein in the developing mouse incisor in wild type mice. **(A)** Eda expression was detected using immunohistochemistry in the dental lamina (DL) as well as in the vestibular lamina (VL) in the anterior area of the mandible at ED12.5. **(B,C)** At later stages, Eda protein was detected in the anterior superficial area of the oral epithelium interconnecting the tooth germ with the VL, at the base of the incisor germ (asterisk). **(B′,C′)** No Eda protein expression was detected in the functional incisor germ (IG). Enamel knot (EK) detectable at ED14.5 was Eda negative. Eda expression was detected also in the adjacent vestibular lamina externally to the dental epithelium at more advanced stages of the development **(B′,C′)**. In contrast to Eda, Shh protein expression was detected using immunohistochemistry at the labial side of the dental thickening between DL and VL in the anterior area of the mandible at ED12.5. **(D)** Shh protein expression is typically absent in the anterior region at the base of the incisor germ at ED14.5 **(E)**. However, Shh expression is detectable in the enamel knot of the incisor germ and it is already expanding in the inner dental epithelium at this stage of the development **(E′)**. Schematics shows location of histological sections **(B,B′,C,C′,E,E′)** in the incisor regions of more advanced mandibles. Dotted line delimits the epithelium in the sections. Bar indicates 100 µm.

To determine how Eda and Shh might interact, the temporo-spatial expressions of Eda and Shh proteins were assessed in the incisor area of wild type mice (28 embryos were used for Shh expression detection and 26 for Eda) (Figure 1).

**FIGURE 2:**
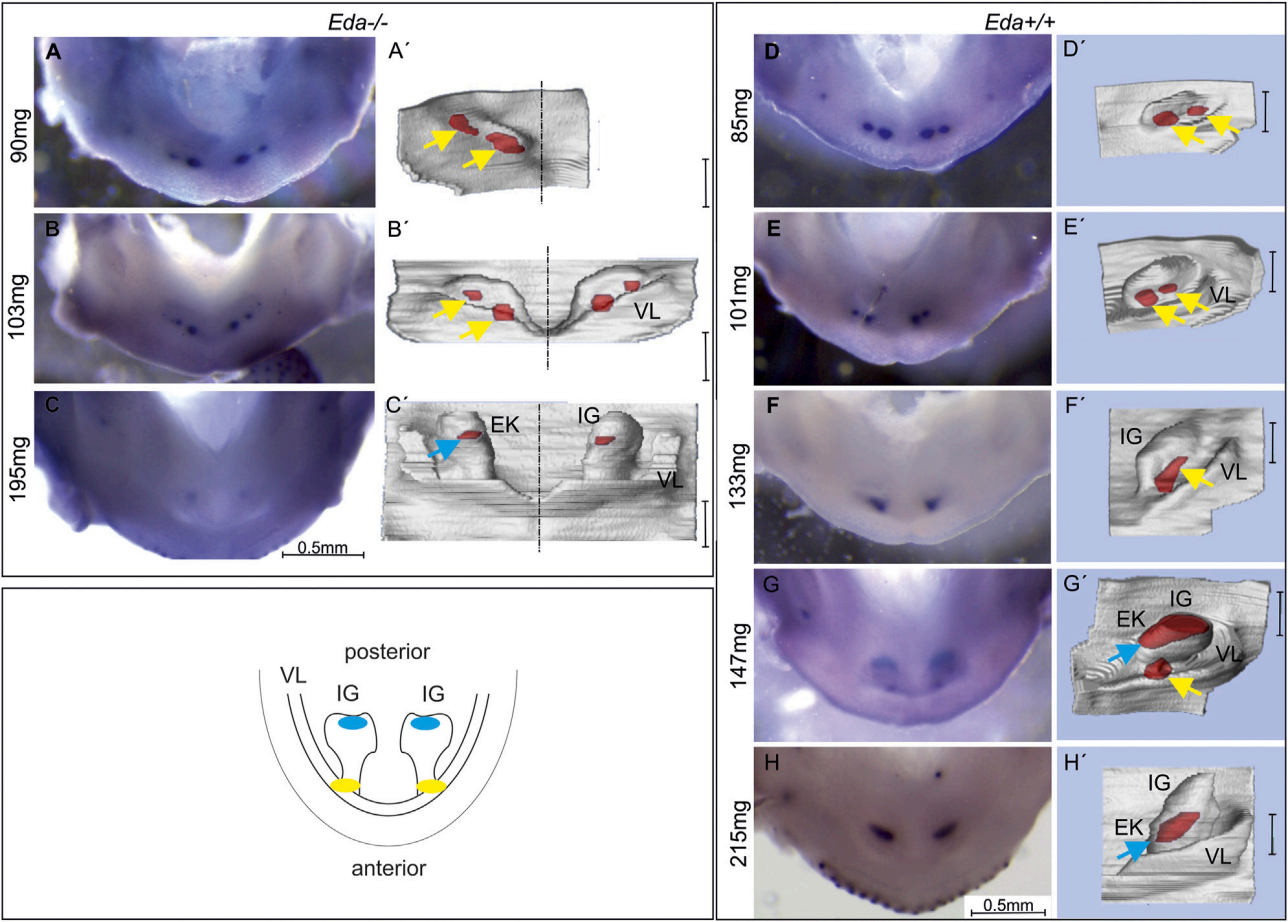
*Shh* expression in the lower incisor area of *Eda−/−* and *Eda+/+* embryos. The expression was visualised by WISH **(A–H)** correlated with morphology of the developing dental and adjacent oral epithelia on 3D reconstructions of the same sample **(A′–H′)**. The schematics shows the incisor region of the WT mouse mandible with developing incisor germ (IG). The anterior early initiation Shh expression (yellow) is located in the region of the epithelium adjacent to the externally invaginated vestibular lamina (VL). This region has been related to the rudimentary incisor generation present as a remnant of additional incisors apparent in the former ancestors (Hovorakova et al., 2011, 2013). The subsequently appearing posterior Shh expression (blue) is located in the region of prospective enamel knot in the functional incisor germ. In *Eda−/−* embryos at ED13.5, anterior, early superficial *Shh* expression region (yellow arrow) split into two spots was detected **(A,A′,B,B′)**. In the more advanced embryo *Eda−/−* (ED14.5, 195 mg) the posterior expression region (blue arrow) was detected in the functional incisor bud **(C,C′)**. In *Eda+/+* embryos at ED13.5, anterior, superficial initiation *Shh* expression localised in two spots in each jaw quadrant was detected **(D,D′,E,E′)**. In the developmentally less advanced embryo *Eda+/+* at ED14.5 (133 mg), only one initiation *Shh* expression region was detectable in the anterior part of incisor area **(F,F′)**. In the more advanced embryo at ED14.5 (147 mg) anterior and posterior *Shh* expression regions co-existed for a short period **(G,G′)**. The posterior Shh expression area (related to the appearing enamel knot (EK) of the functional incisor) was detected on the tip of functional incisor bud **(G′)**. In the developmentally most advanced embryo (215mg, ED15.5) only the posterior *Shh* expression remained visible **(H,H′)**. Bar in 3Ds indicates 100 µm.

**FIGURE 3:**
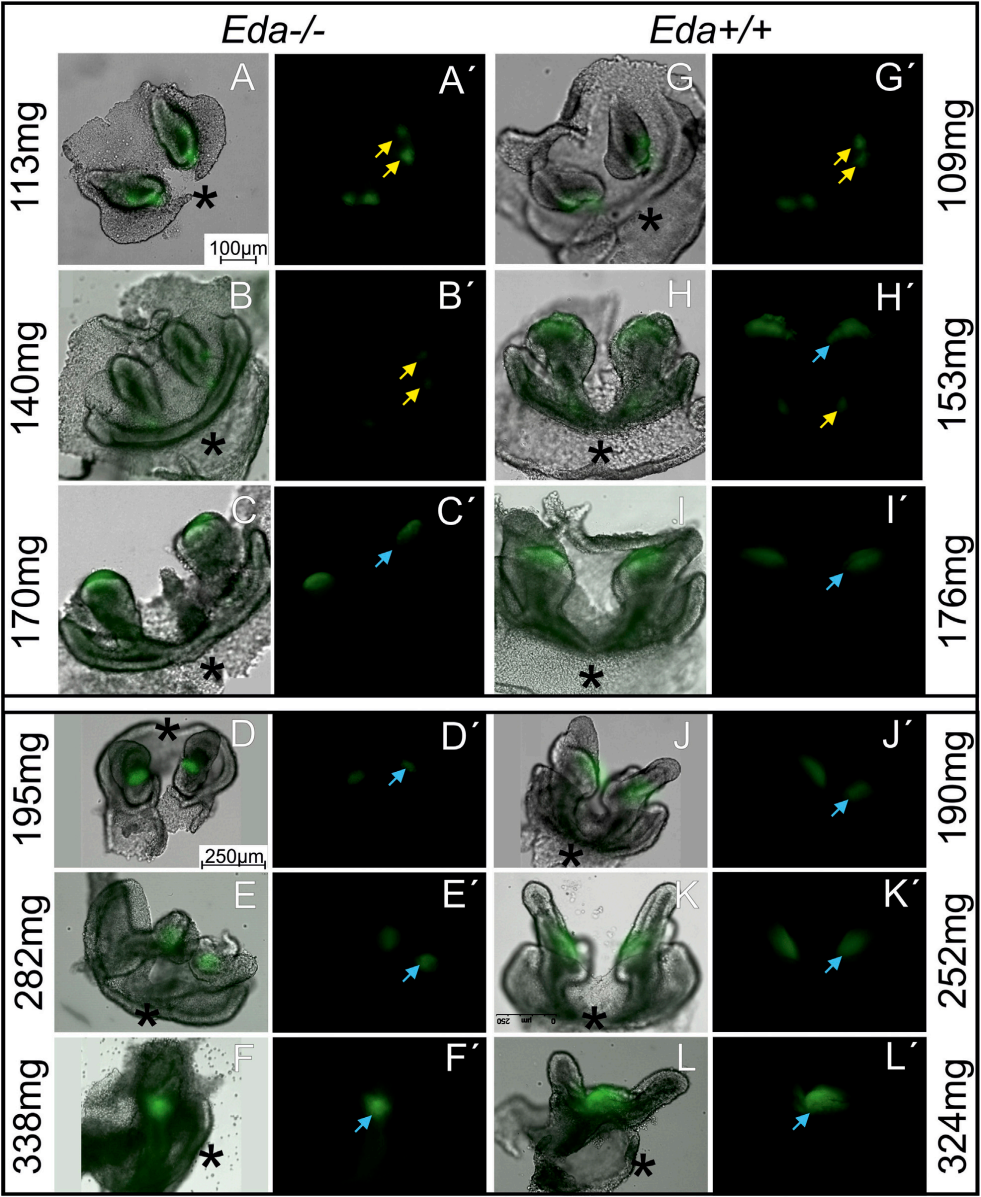
The morphology and Shh expression in the developing lower incisor area in *Eda−/−* **(A–F′)** and *Eda+/+* **(G–L′)** embryonic mandibles. *Shh* expression regions of rudimentary (yellow arrow) and functional (blue arrow) incisor primordiums (see also schematics in Figure 2) are visualised using green fluorescence on the dissociated dental and adjacent epithelia of the incisor area in the *Eda−/−* embryos **(A–F)** and using green fluorescence in the dark field image **(A′–F′)** at ED 14.3 **(A,A′,B,B′)**, at ED 14.7 **(C,C′)**, at ED15.5 **(D,D′,E,E′)** and at ED15.7 **(F,F′)** and of *Eda+/+* embryos **(G–L′)** at ED13.5 **(G,G′,H,H′)**, at ED14.5 **(I–K′)** and at ED 15.3 **(L,L′)**. *Eda−/−* embryos at ED15.5 **(D–E′)** correspond developmentally to the ED14.5 in *Eda+/+* **(J–K′)** and *Eda−/−* embryos at ED15.7 **(F,F′)** to 15,3 *Eda+/+* **(L,L′)**. The development is approximately 1 day delayed in *Eda* deficient mice (see also Supplement 1). The epithelium of the incisor germs in *Eda−/−* **(C–F′)** embryos seemed to be retarded in the growth. The cervical loops of the incisor germs in *Eda−/−* **(C–F′)** embryos were shorter compared to corresponding stages of *Eda+/+* embryos **(I–L′)**. Asterisks determine the anterior direction in the photographs.

Eda protein was detected in the epithelium of the early anterior mandible area associated with the superficial initiation signalling centre at early stages of tooth development (ED12.5) (compre Figures 1A, D). Interestingly, at ED14.5 and ED15.5, Eda was still detectable in this anterior superficial area (Figures 1B, C). In contrast, there was no Eda protein expression detected more posteriorly in the functional incisor tooth germ and in its enamel knot (Figures 1B′, C′), a region where Shh is robustly expressed (Figure 1E′), as shown also using WISH (see Figure 2) and by fluorescence microscopy (see Figure 3). Outside the incisor region, Eda expression was detected labial to the tooth germ in the adjacent epithelial vestibular lamina, the anlage of the oral vestibule (ED12.5–15.5) (Figure 1).

### Different effects of loss of Eda on Shh expression

To assess the impact of Eda deficiency on Shh expression, *Eda−/−* and control *Eda+/+* mice were collected and the expression of Shh compared by WISH (in 93 *Eda−/−* and 57 *Eda+/+* mouse embryos). The expression of *Shh* is extremely dynamic over time and therefore stage matching of embryos is crucial for comparison. *Eda−/−* were smaller than *Eda+/+* on the same background, suggesting an overall delay in development (Supplementary Image S1). As body-weight has been shown to reflect the successive stages of tooth development (Peterka et al., 2002), embryos were compared by weight rather than chronological age (Figure 2).

Using WISH, *Shh* expression was apparent anteriorly at ED12.5 in the initiation signalling centre in both *Eda−/−* (80 mg) and control *Eda+/+* (85 mg) mandibles (Supplementary Image S2). The Shh expressing region was split medio-laterally into two small areas in *Eda−/−* (Figures 2A–B′) and *Eda+/+* embryos (Figures 2D–E′)**.** The anterior Shh signalling region remained detectable until ED13.5 (body weight 103 mg) in *Eda−/−* (Figure 2B, B′) and in control *Eda+/+* (body weight 147 mg, Figure 2G, G′) mandibles*.* The newly appearing *Shh* expression in the enamel knot of the incisor was detectable from ED14.5 (body weight of 195 mg in *Eda−/−* and 147 mg in *Eda*+/+) more posteriorly in the mandible (Figures 2C, C′, G, G′). In contrast to *Eda−/−*, a transient co-existence of the initiation *Shh* signalling centre with the posterior signalling centre of the incisor enamel knot was detectable in *Eda+/+* mandibles (Figures 2G, G′, embryo of 147 mg). This *Shh* expression pattern of co-existing antero-posterior signalling centres activity was not detectable in Eda deficient mice during the whole period of observation. The appearance of the posterior *Shh* expression was also substantially delayed in *Eda−/−* (Figures 2C, C′, body weight of 195 mg in *Eda−/−*) when compared to the controls (Figures 2G, G′, body weight of 147 mg in *Eda+/+*). Interestingly, *Shh* expression in the posterior, and later appearing signalling centre in *Eda*−/− mandibles also seemed to be reduced in its intensity and size when compared to *Eda+/+* embryos (compare Figures 2C, C′, H, H′).

Histological analysis and 3D reconstructions confirmed that the *Shh* expression in the initiation signalling centre was located more superficially in comparison to the more posterior Shh signalling region in both *Eda−/−* and *Eda+/+* mice (compare Figures 2B′, C′, G′).

### Visualisation of the epithelium highlights that *Eda−/−* incisor germs show abnormal morphology at more advanced stages

To provide a more detailed analysis, we moved to *Eda−/−;Shh*EGFP+ and *Eda+/+;Shh*EGFP + mice (112 *Eda−/−* and 109 *Eda+/+* mouse embryos) to follow the pattern of Shh by expression of green fluorescent protein (GFP). To reveal the regions of Shh expression, the surrounding mesenchyme was removed to view the epithelium in isolation. Dissociation allowed the precise shape of the tooth germ to be revealing and compared with the green fluorescent protein (GFP) deep in the epithelial tissue, since the thickness of the mandible limits GFP detection using fluorescence microscopy. This approach enabled us to determine the exact location of Shh expression in the developing epithelium of the tooth germs (Figure 3).

The shape of the dental epithelium as well as the pattern of Shh expression was analysed. At body weights lower than 140 mg (ED13.5 in *Eda+/+* and 14.3 in *Eda−/−*), a signalling centre was present in the early incisor germ which was split medio-laterally into two small areas in both *Eda−/−* and *Eda+/+* embryos (Figures 3A, A′, B, B′, G, G′)**.** More posteriorly, a new Shh expression region appeared directly in the arising functional incisor bud in *Eda+/+* mandibles of embryos with body weight 153 mg (ED13.5, Figures 3H, H′), co-existing with the anterior expression, what reflected the situation observed in WISH samples (Figures 2G, G′). The first superficial Shh expression started to disappear at the same stage in *Eda+/+*. In *Eda−/−* specimens the posterior signalling centre started to be detectable as late as in embryos of body-weight 170 mg (ED14.7, Figures 3C, C′). The transient co-existence of antero-posterior signalling in both regions was not detected, similarly to what observed in WISH samples. In both *Eda+/+* and *Eda−/−* specimens of body weight about 170 mg (ED14.5 resp. 14.7) and in developmentally still more advanced specimens, only the posterior Shh expression region in the enamel knot of the functional incisor germ remained detectable. This posterior signalling was less intense and smaller in size in *Eda−/−* mandibles compared to control *Eda+/+* (compare Figures 3D–F′, J–L′).

**FIGURE 4:**
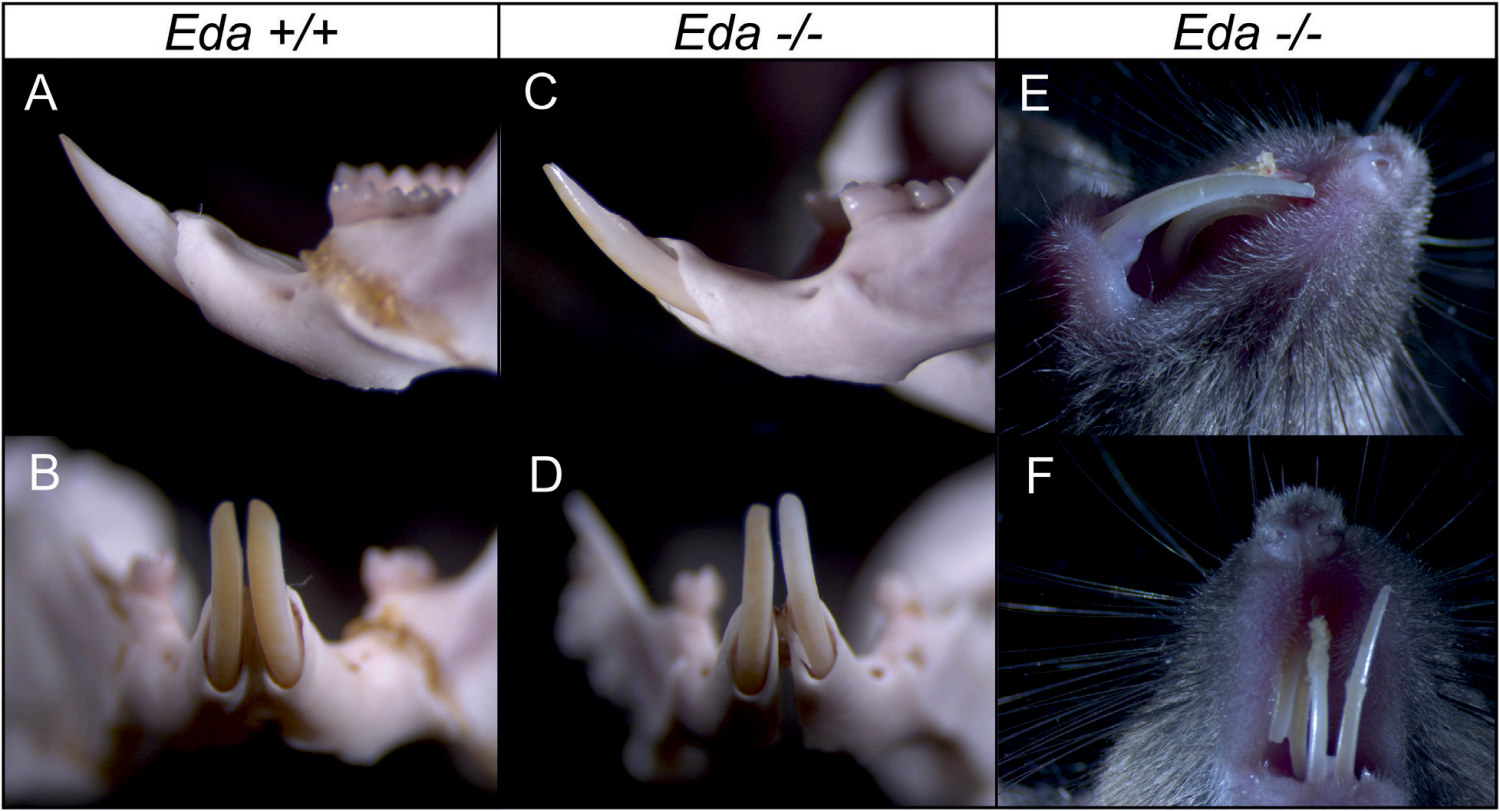
Incisors in the adult control *Eda+/+* and *Eda−/−* mice. Compared to control *Eda+/+* mice **(A,B)**, the erupted incisors in *Eda−/−* mice show variable phenotype from only slight hypoplasia **(C,D)** to hypomineralized and hypoplastic incisors **(E,F)** what fits with previous analysis (Miard et al., 1999; Risnes et al., 2005).

The shape of the dental epithelium of tooth germs was very similar in less advanced stages (until body weights of 170 mg) in both *Eda−/−* and *Eda+/+* embryos. However, in more advanced stages, the epithelium of the cervical loops of the incisor germs was much shorter in *Eda−/−* embryos compared to *Eda+/+* (embryos of 170 mg and more, Figures 3C, C′, I, I′, D–F′, J–L′). Adult *Eda−/−* mice had surprisingly well developed incisors but with very variable phenotypes, with some mice displaying hypoplasia and/or hypomineralization (Figure 4), agreeing with previous analysis (Miard et al., 1999; Risnes et al., 2005).

### The descendant cells of the *Shh* expressing populations contribute to a reduced area in *Eda−/−* incisors

**FIGURE 5:**
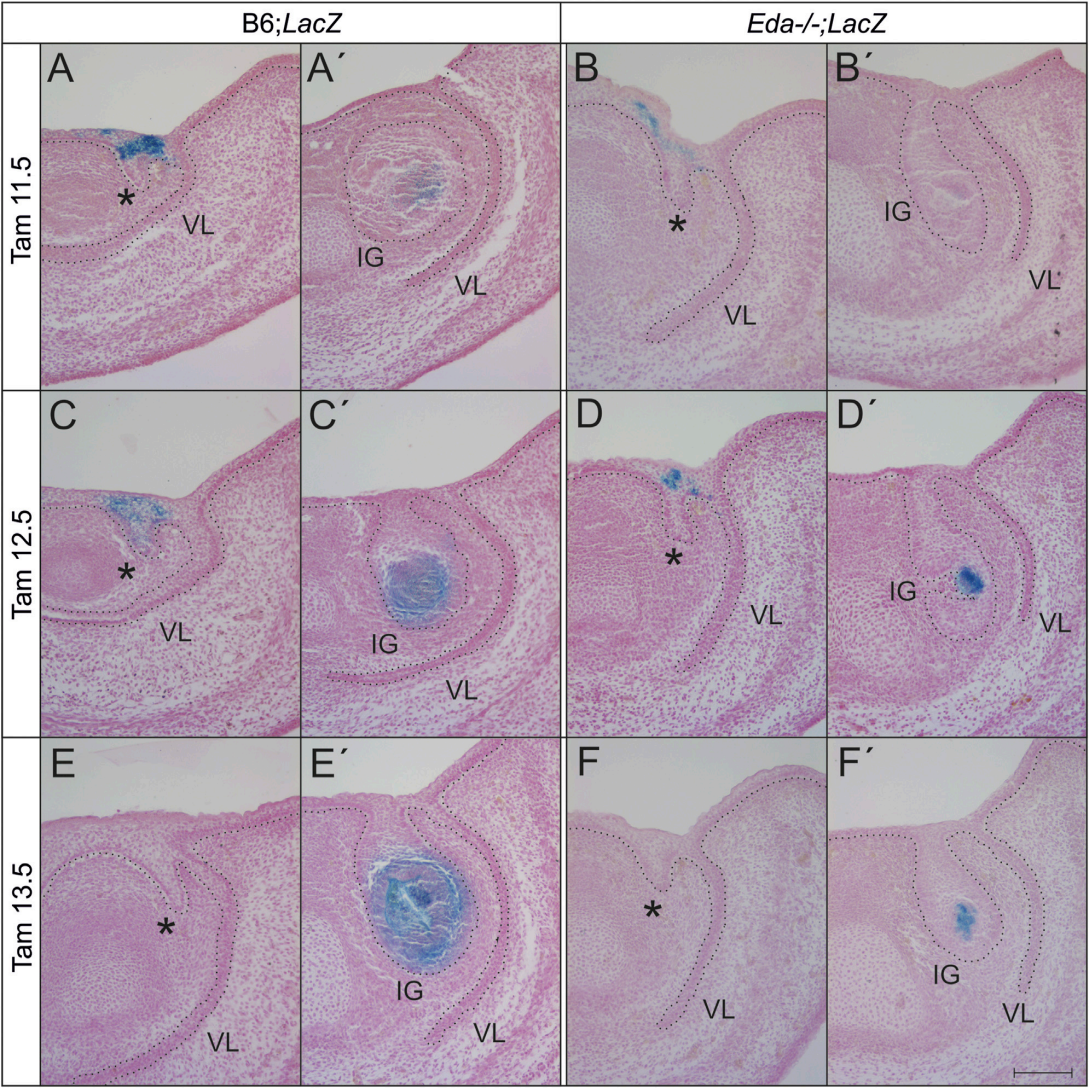
The area of presence of the descendants of cell lineage expressing *Shh* at ED11.5, 12.5 or 13.5 and later is reduced in *Eda* deficient incisor germs at ED14.5 and 15.5. After the tamoxifen administration at ED11.5 and 12.5, the anterior superficial area (asterisk, rudimentary incisor related) of X-gal positive cells (blue) appears to be similar in both *Eda−/−* (*Eda−/−;LacZ*) and WT (B6;*LacZ*) embryonic lower incisor germs at ED14.5 **(A,B)** and 15.5 **(C,D)**. However, the posterior area of positive cells, directly in the tooth germ (IG) of the prospective functional incisor is considerably reduced in space in *Eda* deficient mice (*Eda−/−;LacZ*) at the same stages **(A′–D′)**. After the tamoxifen administration at ED13.5, the anterior superficial oral epithelium adjacent to the tooth germ as well as in the epithelial stalk of the incisor germ was negative in both *Eda−/−* and controls **(E, F)** in contrast to the descendant cell population of the enamel knot area of incisor germ, which was in *Eda−/−* embryos significantly reduced **(F′)** compared to controls **(E′)**. VL–vestibular lamina. Dotted line delimits the epithelium. Bar: 100 µm.

To follow the contribution of the Shh cells during later development, the Shh expressing population was lineage traced in both *Eda* mutant (N = 8) and wildtype (N = 58) embryos using ShhErt2creLacZ mice. The size of the descendant area of Shh expressing cells in the enamel knot was reduced in *Eda* deficient mice compared to control LacZ mice in both experimental groups (embryos harvested at ED14.5 and 15.5 after tamoxifen administration at ED11.5 and ED12.5 (Figure 5)). Interestingly, the anterior area of X-gal positive cells marking the descendants of the initial *Shh* signalling centre located in the anterior and superficial incisor region was similarly spread in *Eda−/−* and controls, agreeing with the similar expression of Shh in these regions at earlier stages. X-gal positive cells were present in the superficial oral epithelium adjacent anteriorly to the tooth germ as well as in the epithelial stalk of the incisor germ in both *Eda−/−* and controls. After the tamoxifen administration at ED13.5, when the anterior signalling centre stops its activity and only posterior signalling centre is expressing Shh, the descendant cell population in the enamel knot area of incisor germ in Eda −/− embryos was also significantly reduced compared to controls (Figure 5). The anterior area in the superficial oral epithelium adjacent to the tooth germ as well as in the epithelial stalk of the incisor germ in both *Eda−/−* and controls was negative in the specimens after tamoxifen administration at ED13.5 (Figure 5).

### The overall level of Shh protein in the lower jaw is not reduced in Eda deficient mice

**FIGURE 6:**
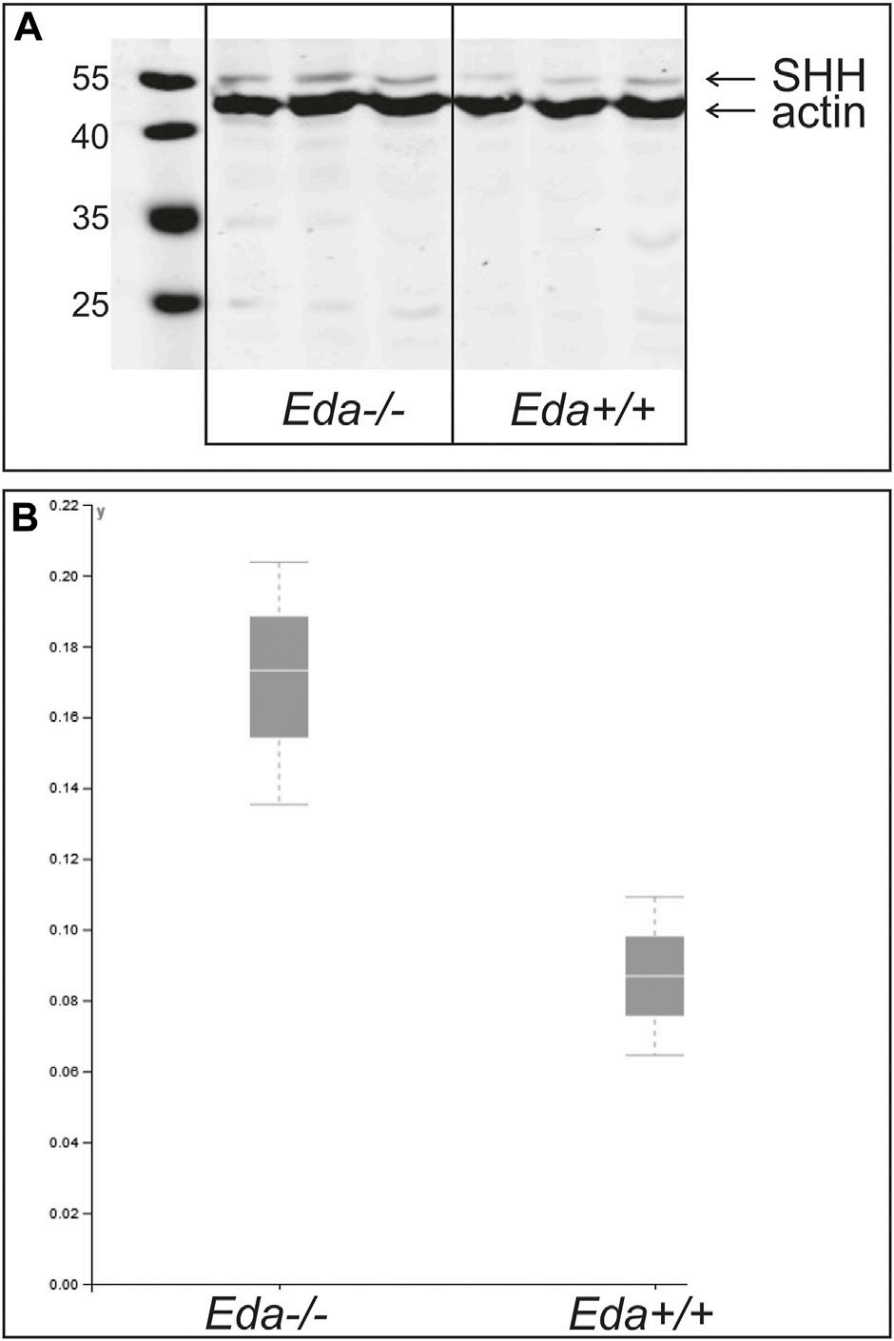
Relative amount of Shh protein at ED14.5 in *Eda−/−* and *Eda+/+* mice. A representative WB experiment of three independent analyses is shown in the image **(A)** and graph **(B)**. Resulting images were scanned and protein quantification was performed using VisionCapt software. Intensity of bands was quantified normalized to actin control. The analysis was performed in three independent harvestings. The normality of the data was tested by Shapiro-Wilk test and consequently One Way ANOVA test was used to determine the differences of the relative protein levels in *Eda−/−* compared to control *Eda+/+*. The differences of relative amounts of Shh protein quantified to the actin levels were not significant.

To assess whether less Shh protein was produced in the jaws of Eda deficient mice, Shh protein levels in the lower jaws were compared at ED14.5 by western blot using 9 *Eda−/−* and 5 *Eda+/+* embryos. At this stage the expression of Shh in the anterior initiation signalling centre has ceased in *Eda−/−* as well as in WT mice (see Figures 2, 3). The expression of Shh at this stage is found in the molar and incisor enamel knots and developing whisker follicles on the chin (Iseki et al., 1996). The tongues were removed to exclude impact from Shh in the tongue. Interestingly, the differences in relative amounts of uncleaved Shh protein produced in *Eda* deficient and control mice were not significant, indicating that despite the Shh signalling region being smaller in mutants the amount of Shh protein produced was unaffected (Figure 6).

## Discussion

This study analysed the early development of the lower incisor region in *Eda*−/− mice and the role of Eda protein during the development of the mouse incisor. The differences in the formation of two subsequently appearing signalling centres, the anterior early initiation signalling centre and the more posterior later enamel knot signalling centre, was focused on by following the temporo-spatial dynamics of Shh expression. Phylogenetically, these signalling centres have been related to the signalling centres of a rudimentary tooth germ and functional incisor, respectively (Hovorakova et al., 2011, 2013).

### Expression of Shh in the early initiating signalling centre appears normal in the absence of EDA protein in contrast to the enamel knot signalling centre in the incisor germ

In the present study we show that the early *Shh* expression pattern in the anterior and superficial area representing a rudimentary incisor generation was not altered in *Eda−/−* embryos (see Figures 2, 3). Thus, the *Shh* expression pattern in the superficial epithelial thickening during early incisor development in *Eda−/−* specimens was comparable to *Eda+/+* (compare Figures 2A, A′, B, B′, D, D′, E, E′) (Hovorakova et al., 2011). This was perhaps unexpected as previous analysis of *Eda* mutants has shown that a slight decrease in the volume of the early signalling centre and a reduction in the number of non-proliferating (G1) cells (Ahtiainen et al., 2016). In contrast to the presence of Shh in the early signalling centre, the later forming more posterior and deeper *Shh* expressing region of the enamel knot of the prospective functional incisor was clearly reduced in size and its appearance was delayed in *Eda−/−* embryos (compare Figure 2B, B′ with Figure 3H, H′, resp. Figure 2G, G′).

As we showed using immunohistochemistry, Eda is expressed during early stages of the development of the incisor germs in the odontogenic epithelial thickening at the same stage when the initiation signalling centre is formed. However, its expression was not detectable later in the development in the epithelium of the functional incisor tooth germ (Figure 1). The expression of Eda in the early signalling centre agrees with the findings using NFkB-LacZ reporter mice (Ahtiainen et al., 2016).

Interestingly, it has been shown that *Eda* plays a key role in the formation of ectodermal placodes acting as signalling centres during the initiation of organogenesis of ectodermal organs (Mustonen et al., 2004). It has been also shown that signalling by Eda protein regulates the size of the ectodermal signalling centres of different ectodermal appendages (Pispa et al., 1999; Mustonen et al., 2004). In the mouse incisor the early Eda expression appears to play a role in control of the primary enamel knot at later stages of the functional incisor. It has been proposed that Eda-Edar mediating the signal is not involved in the induction of the signalling centres but rather in regulation of their function (Laurikkala et al., 2001). In the dental epithelium, *Edar* is expressed in *Eda* negative cell population forming signalling centres. Eda acts as a planar signalling within the epithelium and by binding to its receptor Edar it regulates the functions of the signalling centre of the tooth–the enamel knot (Laurikkala et al., 2001).

As mentioned above, the enamel knot of the incisor germ is Eda negative (Figure 1). Eda expression was limited to the superficial area in the early anterior mandible, which represents the remnant of the rudimentary incisor generation in WT (CD1) mice (see Figure 1, Hovorakova et al., 2011).

It has been shown previously that *Eda-A1-Edar* signalling stimulates the formation of ectodermal placodes in ectodermal derivatives such as hair, teeth and mammary glands (Mikkola and Thesleff, 2003; Mustonen et al., 2004). However, *Eda-A1* deficiency did not affect the initiation of the first forming placodes in any of the organ systems (Mustonen et al., 2004). In the molar teeth the formation of smaller enamel knots has been experimentally induced by adding a soluble form of Edar to tooth germs demonstrating the involvement of endogenous Eda in molar tooth development (Tucker et al., 2000) and mimicking the situation observed in Tabby mice, where the enamel knot size is reduced in molar teeth (Peterkova et al., 2002). Interestingly, it was not possible to reproduce the downless cap stage phenotype (Tucker et al., 2000).

Taken together the early initiation signalling centre related to the rudimentary incisor generation in the mouse seems to be *Eda* independent as well as the initiation of the later enamel knot signallings centre. However, as we show here during incisor development, Eda plays a role in the restriction of the enamel knot centre and its size.

### Loss or reduction of EDA protein expression could be responsible for increased lateral inhibition

It appears that the formation of the enamel knot of the functional incisor is delayed in *Eda−/−* (compare Figures 3C, C´ (170 mg *Eda−/−*), with Figures 3H, H′ (153mg, *Eda+/*+)). In CD1 mice, two antero-posteriorly located successional signalling centres in the anterior mandible have previously been shown co-expressing *Shh* for a short transient time period (Hovorakova et al., 2011). In *Eda+/+* control mice, the expression of Shh fully reflected the situation shown in CD1 mice and the co-expression of both signalling regions was detected (Figures 2D–H′; Figures 3G–L′). However, in *Eda* deficient mice the transient co-existence of the diminishing anterior initiation *Shh* signalling centre and newly appearing posterior enamel knot of the incisor was not detected (Figures 2A–C′). The posteriorly appearing Shh protein expression corresponding to the enamel knot of the functional incisor germ also appeared to be less intense and smaller in size when compared to the situation with control *Eda+/+* mandibles (Figures 3F, F′, L, L′). To precisely determine the area of the *Shh* expressing cells in the enamel knots of incisors in *Eda* deficient and control mandibles, the tamoxifen inducible Cre-loxP system was used. This allowed the tracing of *Shh* expressing cells and all their descendants after the tamoxifen injection. The results clearly showed that all the cell descendants of the population expressing *Shh* in enamel knot in the *Eda* deficient incisor germs take up substantially reduced space when compared to the relevant stages in control incisor germs (Figure 5). Interestingly, it has been shown that morphologically the enamel knot structure was markedly reduced in the molar tooth germs in *Eda−/−* mandibles (Peterkova et al., 2002).

It has been suggested that the increased *Eda-A1* affects the balance of activators and inhibitors, and impairs the lateral inhibition mechanism responsible for both the expansion of the placodes and initiation of successive placodes (Mustonen et al., 2004). The observations reported here suggest that the deficiency of *Eda* might be responsible for an increased lateral inhibition mechanism. This would lead to the impaired and spatially reduced *Shh* expression in the enamel knot of the incisor in *Eda−/−* mice. As the consequence the cervical loops of the incisor germs of *Eda−/−* embryos were much shorter at more advanced stages compared to controls (Figures 3C–L′; Figure 5). The adult *Eda−/−* mice had well developed incisors but showed signs of hypoplasia and/or hypomineralization (Figure 4). Previous morphological observation also reported hypoplasia, shortening and thinning of affected incisor enamel organs in 100% of *Eda−/−* mouse embryos (Miard et al., 1999). Interestingly, our WB results show that the reduction of the size of the signalling centres in *Eda* deficient mice was not accompanied by a significantly reduced levels of Shh produced (Figure 6). In contrast, the levels of non-cleaved Shh appeared to be higher in *Eda−/−* embryos than in controls of corresponding stages of development. The difference however was not significant. Interestingly, although the adult erupted incisors are slightly hypoplastic they are only rarely severely affected in *Eda−/−* mice. This fact shows that tooth development is complex and members of different signalling pathways may be able to partially compensate their roles as previously suggested for this pathway (Tucker et al., 2000), which may be supported by morphological observation (Lesot et al., 2004).

It seems that Eda is essential during very early stages of functional incisor development but the effect of its absence on the tooth germ can be partially compensated during later development. Further investigation will be necessary to reveal how spacing of signalling centres influences Shh production, and how such changes produce the wide range of dental pathologies observed in Eda mice.

## Conclusion

*Eda* deficiency led to delayed and decreased *Shh* expression from ED13.5 resulting in hypoplasia of the functional incisor primordium at more advanced developmental stages. We propose that, *Eda* deficiency leads to increased lateral inhibition responsible for a reduction in the size and spacing of the enamel knot signalling centre in the lower incisor germs.

To summarize our results:

1) Eda protein expression in the mouse incisor region is limited to the anterior superficial area representing the rudimentary incisor generation and initiation signalling centre.
2) The early region of Shh expression in the superficial incisor region appears unaffected in *Eda* deficient mice.
3) The later region of *Shh* expression in the enamel knot of the functional incisor is delayed and reduced in size in *Eda* deficient mice.

Based on this we suggest that the *Eda* signalling could play a role in the space restriction of the signalling centre of the successional tooth during tooth development. Further investigation will be needed to determine the precise role of *Eda*, as well as to elucidate the exact activation mechanisms for Edar in this process.

## Data Availability

The original contributions presented in the study are included in the article/Supplementary Material, further inquiries can be directed to the corresponding author.
