# Supplementary figures and images for: Eda controls the size of the enamel knot during incisor development
Source: Front Physiol. 2023 Jan 9;13:1033130. doi: 10.3389/fphys.2022.1033130 (PMC9868551; doi:10.3389/fphys.2022.1033130)

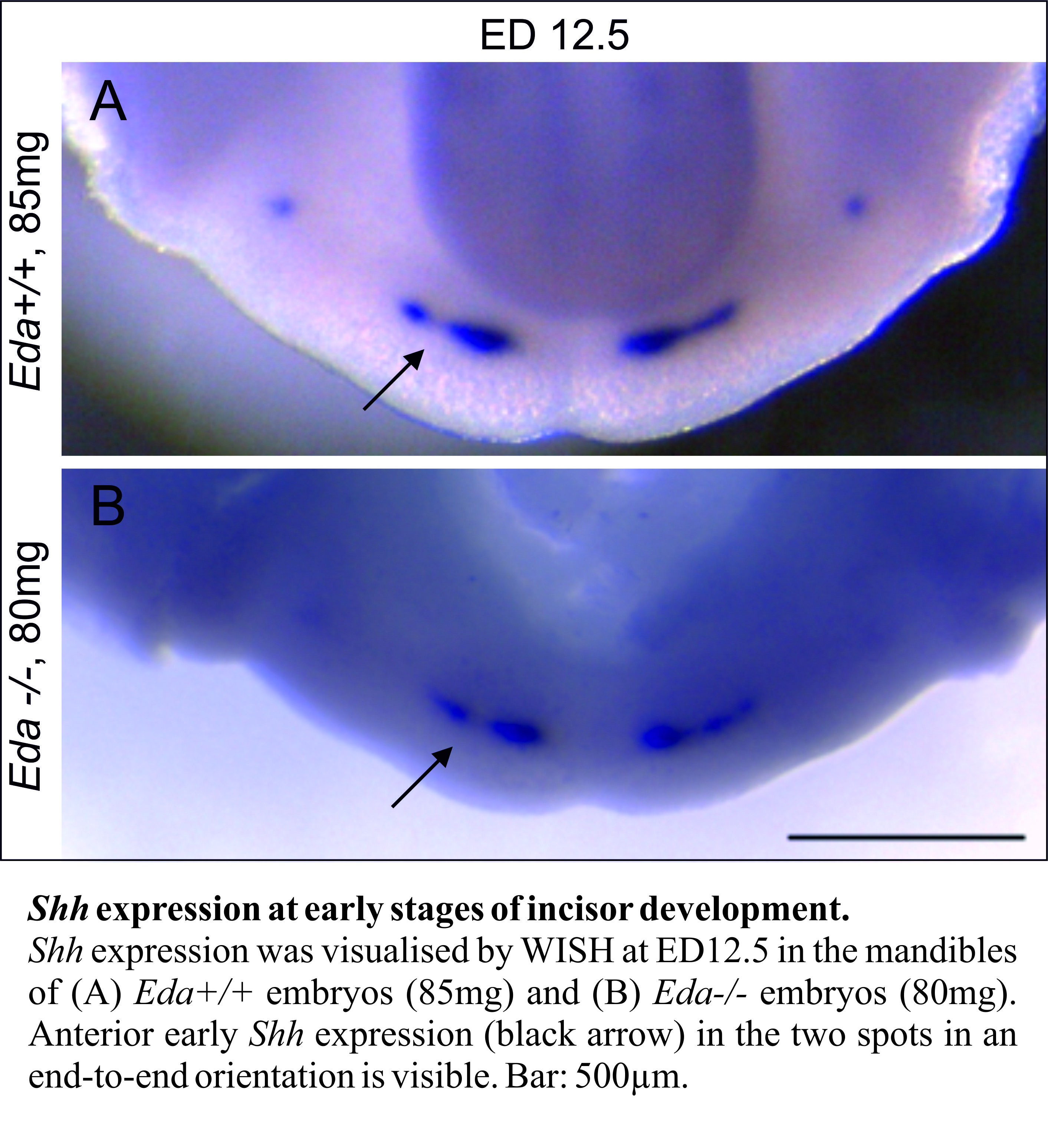

Supplement: Supplementary file 1 [file Image2.jpg]

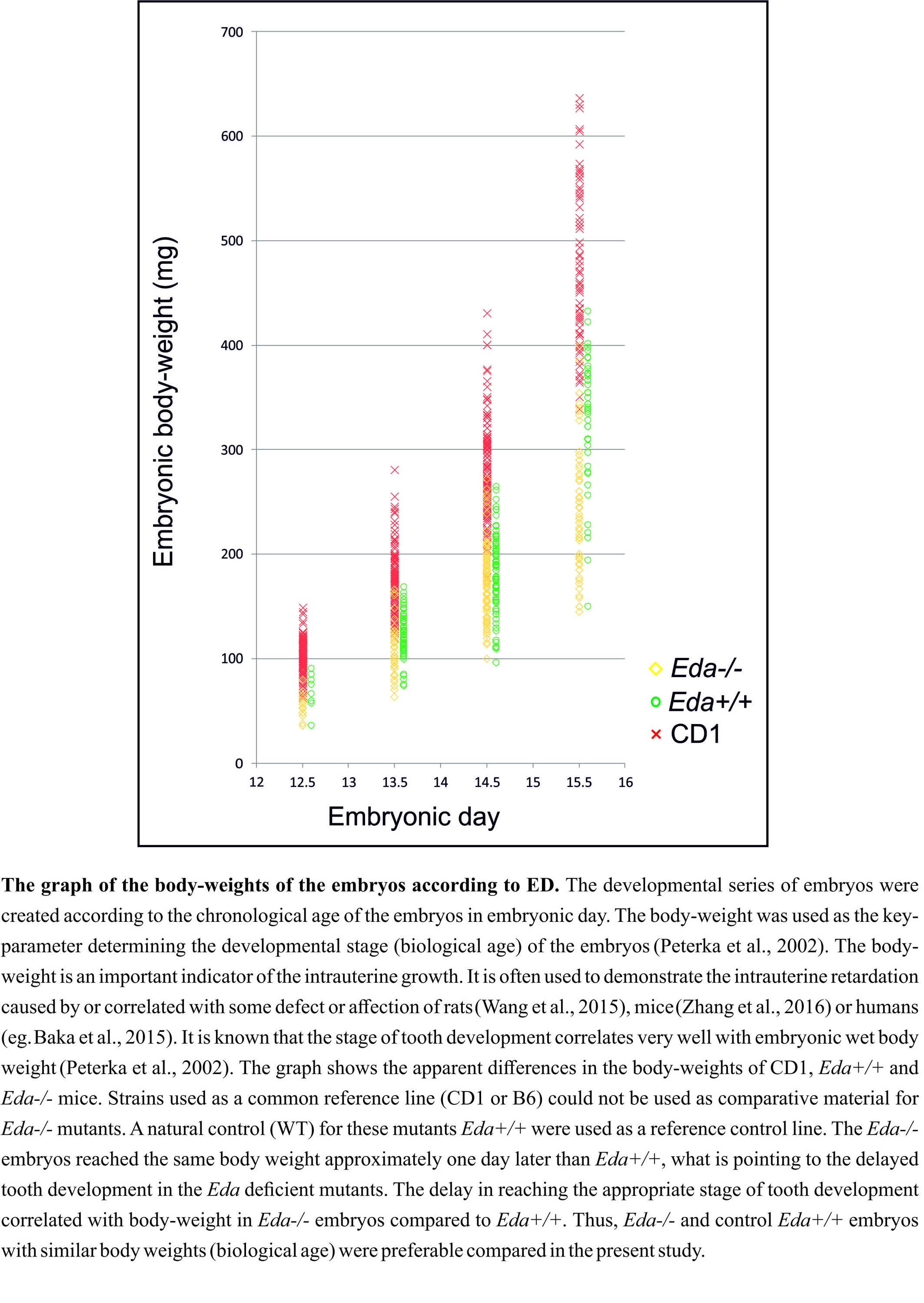

Supplement: Supplementary file 2 [file Image1.jpg]
